# Expectant mothers and fathers' knowledge of nurturing care in a developing country

**DOI:** 10.3389/fped.2022.1024593

**Published:** 2022-11-22

**Authors:** Hafize Soysal Cimen, Bedriye Tugba Karaaslan

**Affiliations:** ^1^ Child Development Master's Program, Institute of Health Sciences, Üsküdar University, Istanbul, Turkey; ^2^ Department of Child Development, Faculty of Health Sciences, Izmir Katip Celebi University, Izmir, Turkey

**Keywords:** nurturing care framework (NCF), early childhood, pregnancy, expectant mothers and fathers, child development

## Abstract

**Background:**

The Nurturing Care Framework (NCF), which has been emphasized by the World Health Organization (WHO) in recent years and is among the United Nations Sustainable Development Goals (SDGs), expresses the conditions created to promote early childhood development (ECD). These conditions aim to provide opportunities for children in the Good Health, Adequate Nutrition, Responsive Caregiving, Security and Safety, and Opportunities for Early Learning (5 Components) by caregivers, primarily from low and middle-income countries (LMICs). The aim of this study is to examine the knowledge and awareness of expectant mothers and fathers in Turkey, about the NCF.

**Methods:**

In this study, a cross-sectional, analytical research design was used. Nurturing Care (NC) knowledge and awareness levels of expectant mothers and fathers in the 2nd and 3rd trimesters of pregnancy were determined *via* the online form of “The Knowledge of Nurturing Care Inventory (KNCI)” prepared by the researchers. This form consists of 60 questions describing the 5 components mentioned above. The sample contains a total of 103 people, 91 (88.3%) women, and 12 (11.7%) men.

**Results:**

Among the findings, the highest mean of knowledge level (13.76 ± 1.22) was found in Responsive Caregiving, and the lowest mean of knowledge level (4.35 ± 1.83) was found in Opportunities for Early Learning. The general level of knowledge on NC was found to vary statistically significantly depending on gender in favor of the women [*t*(101) = 3.27, *p* < .05], depending on educational status in favor of those with university and graduate education [*F*(2, 100) = 3.481, *p* < .05], depending on participation in pregnancy school training in favor of those who attended [*t*(101) = 2.349, *p* < .05], and depending on knowledge on how to support development in favor of those who know how to support [*t*(101) = 2.370, *p* < .05].

**Conclusions:**

As a result, expectant parents need information and support about the period when children can acquire the basic developmental milestones and about the developmental risk indicators. More research is needed to implement the NCF starting from the preconception period, especially in LMICs, so that the opportunities in early childhood (which is the time period when the brain architecture is shaped, and development is the fastest) are not missed.

## Introduction

In all circumstances, it is important to inform parents and caregivers about nurturing care (NC) and its components and to raise their awareness levels to support development in all aspects of early childhood, which is one of the most important times of life. This has become even more crucial during the COVID-19 pandemic. In the literature, early childhood is considered to be very valuable, as it is the period in which development is the fastest in many areas and forms the basis for the following periods. The studies examining how early childhood experiences shape the architecture of the human brain emphasize the lifetime impact of these experiences (1, 2). In fact, researchers draw attention to the interaction of ecology, biology, and development through the ecobiodevelopmental approach, and argue that the experiences show their effect by causing changes not only in the current generation but also in the next generations through genetic inheritance (3, 4). The Benchmark study, conducted with a different perspective in Canada, tried to determine the level of knowledge of the families on the issues such as health, care, and responsive caregiving for their children, is one of the studies that guide professionals and focuses on the social impact of these efforts (5).

The healthy growth and development of children, especially in the first 1,000 days of life, depends on the knowledge and resources that parents and caregivers need to be able to provide NC, the most important of all modifiable factors (6). Although the Nurturing Care Framework (NCF), in which not only development but also all the components affecting development are addressed together, and the contributions of this framework to early childhood development (ECD) have come to the fore recently, there is a gap in knowledge regarding the characteristics of the caregiver that contribute to optimal development from pregnancy to birth (7). In the literature there are many international studies (8–16) and national studies (17, 18) aiming to learn especially mothers' level of knowledge about ECD, however, the research investigating expectant parents’ level of knowledge on this subject during pregnancy is limited (19). Thus, this study aimed to determine the knowledge level and awareness of expectant mothers and fathers about NC during pregnancy and to examine their knowledge and awareness in terms of different variables.

The term NCF, which has been strongly emphasized by the World Health Organization (WHO) in recent years, refers to the conditions created by public policies, programs, and services. These conditions allow caregivers to ensure good health, adequate nutrition, and protection from threats to children. While NC also aims to provide young children with responsive caregiving and early learning opportunities, UNICEF and WHO recommend that the NCF be implemented in all countries in order for children to reach their developmental potential (20).

In the United Nations Sustainable Development Goals, the “Nurturing Care Framework” is extensively discussed to promote ECD. In relation to the importance of ECD, comprehensive and up-to-date versions of the early childhood interventions within the context of the following components of the NCF are presented: “Good Health Component/GHC”, “Adequate Nutrition Component/ANC”, “Responsive Caregiving Component/RCC”, “Safety and Security Component/SSC” and “Opportunities for Early Learning Component/OELC” (21).

The basic principles included in GHC, ANC, RCC, SSC, and OELC are expressed in Table 1 (22).

**Table 1:** Basic principles within the scope of the components of nurturing care.

	Basic principles
Good Health	Family planning, prevention and cessation of smoking, alcohol and substance use, prenatal care-birth care, prevention of mother-to-child transmission of HIV, extra care for small and sick babies and basic newborn care, postnatal care, kangaroo care-low birth weight infant care, childhood immunizations, care for children living with developmental difficulties and disabilities, carrying out parent mental health support activities, early detection of illness or disability (e.g., vision, hearing), seeking timely and appropriate care for sick children
Adequate Nutrition	Maternal and infant nutrition, complementary feeding and transition to healthy nutrition in the family, intake of micronutrients when necessary, monitoring of and intervention in growth when necessary, management of all kinds of malnutrition
Responsive Caregiving	Skin-to-skin contact immediately after birth, caring and safe adult care in the family environment, guiding children in daily activities and relationships with others, establishing daily feeding and sleep routines, involvement of fathers and extended family, providing social support
Safety and Security	Meeting the need for safe water, preventing child abuse and neglect, preventing and reducing indoor and outdoor pollution, providing healthy-green, toxins-free environments, preventing spouse and domestic violence, providing safe playgrounds in urban and rural areas
Early Learning	Communicating with children through vocalization, responding with facial expressions and gestures, providing language stimulation through speech and singing, encouraging caregiver-guided exploration of objects, caregiver-child toy, encouraging reading and storytelling, supporting mobile toy and book libraries, quality day care and conducting activities to ensure that every child benefits from preschool education

The existing research has concluded that multicomponent intervention packages should be implemented to make the concept of NC successful and sustainable. The suggestions emphasize that the intervention packages should be implemented at developmentally appropriate times throughout life, target multiple risks, and increase the number of children and families who benefit. Evidence indicates that parents and caregivers must be effectively supported in providing responsive caregiving and protection for young children to reach their developmental potential (21). Data obtained collected in the last decade show that millions of women, children, and adolescents have lagged due to social, economic, and cultural inequalities. The main goal of the strategy determined to solve these problems is to ensure the survival and development of women, children, and adolescents. Because prenatal and early childhoods are the beginning and critical years of human life, all children and their families should be supported in terms of GHC, ANC, RCC, SSC, and OELC, especially during these periods (23).

Although the importance of early childhood is well-known, in a study conducted in LMICs with the participation of 250 million children under the age of 5 (43%), the main causes of the risk of children not to reach their developmental potential were stated to be not using scientific knowledge based on NC that enables progress in children and not being able to take action with multidimensional interventions in the first years of life, which includes critical periods. At this point, it will be instrumental to understand the concept of NC and to elaborate on the person, environment, and stimuli that will provide this care. Nurturing Care is defined as care in which the health and nutritional needs of the child are met, he/she is protected from all kinds of threats, his/her continuous development is supported by giving appropriate stimuli, and the child is provided with opportunities for early learning with emotionally supportive interaction offering responses needed by the child (24, 25).

The NCF provides a roadmap based on recent changes in how child development occurs and effective interventions that can improve ECD. It outlines how parents and caregivers who care for young children can be supported. Nurturing Care recognizes the critical importance of the environment, which is active in all aspects of a child's development, and that the multiple sectors play a role in protecting and supporting brain development throughout life. The framework primarily focuses on children's pre-primary years. It expresses the critical importance of the first 1,000 days, from conception to the end of the second year after birth, when the child's brain development is the fastest, the child's brain is the most vulnerable to harm and the most sensitive to interventions to reduce risks and optimize development. It creates human capital in childhood today, in adolescence and adulthood tomorrow, and in future generations (22).

Data for Turkey were also presented along with 196 other countries within the scope of “Country Profiles for Early Childhood Development”, which was developed by UNICEF in cooperation with the “Countdown to 2030 Women's, Children's and Adolescents’ Health” organization. Turkey's 2021 data regarding the NCF are presented below in terms of each component and its subdimensions. In relation to the GHC, the following values were obtained for its subdimensions: 90% for antenatal care (4 or more visits), 79% for postnatal visits, 45% for care-seeking for child pneumonia, and no data were obtained for treatment for HIV + pregnant women. In relation to the ANC, the following values were obtained for its subdimensions: 71% for early initiation of breastfeeding, 41% for exclusive breastfeeding, and no data were obtained for minimum acceptable diet. In relation to the RCC, it is stated that comparative country data are urgently needed for the subdimensions of public information about ECD, parental mental health, parent support (groups, home visits), and quality child daycare. In relation to the SSC, the following values were obtained for its subdimensions: 100% for basic sanitation, 98% for birth registration, 97% for basic drinking water, and no data were obtained for positive discipline. In relation to the OELC, the following values were obtained for its subdimensions: 76% for playthings at home, 65% for early stimulation at home, 29% for children's books in the home, and no data were obtained for attendance in ECD (26).

The results of the statistics on the NCF in Turkey and the studies on the pregnancy period in Turkey indicate that it should be explained to men and women who have decided to become a family that child development is multidimensional, that many variables affect the development, and that the development process of the unborn baby should begin while in the mother's womb. In a study on the knowledge levels of those who were educated at the pregnancy school and were also pregnant, it was found that the knowledge level of pregnant women increased significantly with the education provided, and those with their first pregnancy and university graduates participated in pregnant education at a higher rate (27).

Expectant mothers and fathers need information on many subjects, such as pregnancy, birth, child health and diseases, and child development. Then, they try to learn this information through various channels. Pregnant education classes in our country have been organized since the 1980s and have become widespread since 2000. Recently, the desire of pregnant women and their husbands to attend childbirth preparation classes has increased. In this increase, the Ministry of Health's support of pregnant women in the direction of vaginal birth, raising awareness of the public through the media and other channels, and the training programs of health professionals working in the field of obstetrics, and their awareness of their duties, authorities, and responsibilities on this issue have been found to be effective (28). Today, prenatal classes aim to prepare expectant mothers and fathers for birth, to provide them with information about pregnancy, birth, postpartum period, and healthy growth of the baby, and to impart knowledge and skills so that they can adopt their new roles (27). The studies carried out in pregnant schools in Turkey mainly focused on reproductive health, preparation for childbirth, birth types, etc (29).; on the other hand, developmental milestones, developmental risks or supporting development, etc., were not considered much. Another study (30) examined the topics that pregnant women researched during their pregnancies. It reported that nutrition, fear of birth, baby development, labor, and coping with nausea were widely researched by pregnant women.

Parents or other caregivers prioritizing the baby's health, growth, care, and development, and preparing themselves and their environment for this process in such a way as to provide NC before the baby is born are considered to be very important investments for the early childhood period. It is very important to determine the health, care, and developmental knowledge levels of expectant mothers and fathers of children between the ages of 0 and 6 to understand their perspective toward the child and the opportunities to be offered to the child. Having information about the level of knowledge possessed in GHC, ANC, RCC, SSC, and OELC, which are the components of the NCF, will inform early childhood professionals in terms of stimuli to be presented to the unborn baby. From this perspective, the study aimed to determine the knowledge levels and awareness of expectant mothers and fathers during pregnancy and to examine them in terms of different variables.

Problem Statement:

• What are the level of knowledge of expectant mothers and fathers of NCF components (GHC, ANC, RCC, SSC, and OELC)?
• What is the general NC knowledge level of expectant mothers and fathers?
• Which variables are significantly correlated with the level of knowledge of NC?

Assumptions:

The assumptions of the study are as follows:

• The sample group selected in the study represents the target population.
• The expectant mothers and fathers who participated in the study, who were in the 4–9 months of their pregnancy, answered the questions asked through the KNCI in a way that best reflected their current knowledge and awareness.
• The expectant mothers and fathers who participated in the study answered the sociodemographic data form in a way that best reflected their current situation.

## Materials and methods

### Research design

The study was planned in a cross-sectional, analytical type of research design to determine the level of knowledge and awareness of expectant mothers and fathers during the pregnancy period. After the participants agreed to participate in the study, they filled out the online form, and the data was collected between November 2021 and March 2022.

### Population and sample of the study

Since the research was carried out during the pandemic period, during the full closure period, since the health directorates were not allowed to collect data in health institutions, and especially the expectant mothers were considered as a risk group during this period, it became necessary to determine the sample and collect data online. Participants were reached by using snowball and criterion sampling methods, which are non-probabilistic sampling methods together. It has been predicted that snowball sampling through social media may introduce sampling bias towards the characteristics of those with an active online presence. In this context, social media statistics were used. When the usage rates of Facebook, Youtube, Instagram and Twitter are examined in the October 2021 update of the report, in which We Are Social and Hootsuite published the digital tables of the countries; It has been reported that Turkey ranks second among countries with a population of over 1 million, with 72.4% Instagram usage for following the agenda (31). Although the number of users is the lowest among these four channels, Twitter stands out as another of the most important channels (68%) for following the agenda. For this reason, the participants of the research were included in the study through these two channels.

57 accounts (accounts run by 31 Midwives, 22 Obstetricians and Gynecologists and 4 Pediatricians) broadcasting in Turkish for prospective parents on Instagram and Twitter were identified and the executives of these accounts were contacted to share the research invitation on their accounts. 28% of the accounts contacted with the sample statements presented below (10 Midwives, 4 Pediatricians and 2 Gynecology and Obstetrics Specialists) shared the research invitation in their accounts. With these accounts, “ *…  'share a research opportunity with expectant parents nationwide’* *… 'We use a snowball sampling strategy through social media sites to reach [to-be parents]’* *… 'We are in the time of the pandemic and any help you can offer to help us reach parents-to-be is invaluable to us’* *… Help us connect with prospective parents’* *… 'We kindly ask you to forward our research invitation to your followers’* *… "We kindly request you to retweet our research invitation for prospective parents”*. After the relevant accounts shared the research invitation, the data were collected by contacting the researchers who agreed to participate in the survey. It is assumed that the survey invitation is seen by all followers of the relevant accounts. Among the followers reached by the invitation, those who were in the 2nd or 3rd trimester of their pregnancy (parents who are in the 4th-9th months of pregnancy) who agreed to participate and contacted the researchers were included in the study. The participants were asked to fill in an online form (Some of the contacted parents candidates were not included in the study at the first stage because they did not reach the 2nd and 3rd trimesters specified as the criteria. However, during the data collection process from these candidates (November 2021 and March 2022) they were not included in the 2nd trimester. Those who passed were included in the study at the time they passed. Although it is impossible to know how many parents-to-be were reached through which social media account or whether they were reached by random chance, these accounts were calculated to have a total of approximately 60,000 members or followers.

Since the number of prospective parents who accepted the research invitation and met the criteria was limited, primigravida condition was not sought, all primigravida or multigravida participants who accepted the invitation and contacted the researchers were included in the sample. As a result;

Inclusion criteria for the research:

• Expectant mothers and/or spouses in the 2nd and 3rd trimester of their pregnancy (primigravida + multigravida)
• Mothers and/or spouses who volunteer to participate in the study
• Expectant mothers and/or spouses who can read and understand Turkish
• Technology literate mothers and/or spouses

Exclusion criteria from the research:

Expectant mothers and/or spouses in the first trimester of their pregnancy.

### Data collection tools

The research data were collected *via* an online form (Google Forms) prepared by the researchers. The online questionnaire form used in the collection of the data was prepared in two parts with the “Google Forms” application and was arranged in a way to respond online. The selection of a response option has been enforced while preparing the online form *via* the Google Forms program. At the same time, the application does not save incomplete surveys. The first part was for obtaining information about the demographic characteristics and working conditions of the expectant parents, and the second section was about the components of NC. The titles of the sections were:

• Socio-Demographic Information Form for the Expectant Parents
• Knowledge of Nurturing Care Inventory (KNCI)

#### Sociodemographic information form

The questions in this form are structured to elicit information about the age, gender, education level, year of marriage, income status of the expectant mothers and fathers, as well as the number of pregnancies, pregnancy loss, gestational age, whether the pregnancy was intentional, pregnancy status, baby's gender, and the participation in pregnant school training. In the determination of the variables to be included in the form, the variables that are commonly associated with pregnancy in the literature were considered.

#### Knowledge of nurturing care inventory (KNCI)

After examining the questionnaires and tools in the national and international literature, the KNCI tool was prepared in Turkish. KNCI was not developed as a scale. Since it is a questionnaire, after it was developed, 25 people, 21 mother candidates and 4 father candidates, were asked to answer the electronical online KNCI questions and give feedback in terms of intelligibility. Necessary adjustments were made, taking into account the feedback given especially on the answer options as well as the question statements. Then, “expert opinion” was taken from 5 field experts (4 faculty members from Child Development Department and 1 faculty member from Department of Developmental Pediatrics) and in line with their suggestions, KNCI took its final form used in the research. The final version of the KNCI was presented in Turkish to the prospective parents forming the sample.

The Knowledge of Nurturing Care Inventory (KNCI) includes the NC Components of Good Health (GHC), Adequate Nutrition (ANC), Responsive Caregiving (RCC), Safety and Security (SSC) and Opportunities for Early Learning (OELC). In the subdimensions of the abovementioned components, there are statements and questions about the care of children in the early childhood period with healthy, safe, and development-supportive approaches. In the process of developing the inventory, a pool was created using many sources and taking into account the clinical experiences. There are 15 questions in each component of the inventory, and it takes approximately 20–30 min to complete the inventory. The Knowledge of Nurturing Care Inventory (KNCI) consists of five components: in GHC, there are 12 true/false questions, 2 multiple choice questions, and 1 sequencing question; in ANC, there are 13 true/false questions, 1 sequencing question, and 1 open-ended question; in RCC, there are 15 true/false questions; in SSC, there are 12 true/false questions, 2 multiple choice questions, and 1 open-ended question. The last component, OELC, consists of 15 questions that question the month range in which the main developmental milestones occur and are coded as correct if the answer is in the correct range and false if not.

### Data collection process

The first step in the data collection process of the study was to take the “Ethics committee permission” of the Üsküdar University Non-Interventional Research Ethics Committee numbered 2021/61351342. After the completion of the legal permission processes, the “Socio-Demographic Information Form” and the “Knowledge of Nurturing Care Inventory (KNCI)” were finalized according to the opinions of the experts.

In the first part of the sociodemographic information form, the participants were asked to read the instructions and give their consent. This text included information about the identity of the researchers, the purpose of the study, its coverage, and the procedures to be used in the study, as well as the information about the ethics committee's permission. In the form of consent obtained from the participants online, it was stated that the study was on a voluntary basis, that the information of the participants participating in the study would not be disclosed in any way, and that the results would not be used for purposes other than scientific research.

Data collection was carried out with the expectant mothers and fathers who agreed to participate in the study between November 2021 and March 2022. The data collection process was intense during the first 18 days of the study, with 58% of the total participants participating in this first period. After the completion of the data collection process, the data were automatic recorded locally using Excel Software and the completed forms were analyzed by the researchers. SPSS (Statistical Package for Social Sciences) 26.0 (IBM Corp., Armonk, New York, United states) was used for the data analysis.

The online cross-sectional study was conducted in accordance with the Checklist for Reporting Results of Internet E-Surveys (CHERRIES) (32).

### Data analysis

As the data collection tools, the “Socio-Demographic Information Form for the Expectant Parents” and the KNCI were used in the study. In the form, there are questions related to sociodemographic characteristics such as gender, age, place of residence, education level, occupation status, information about the pregnancy process, and postpartum baby care. In the inventory, there are five components, GHC, ANC, RCC, SSC, and OELC. The total number of correctly answered questions in the component shows the level of knowledge in that component for each individual. The sum of the knowledge levels obtained by the individual from all the components constitutes the KNCI level. The highest value that can be obtained from the components is calculated as 13 for GHC, 15 for ANC, 15 for RCC, 12 for SCC, 15 for OELC, and 70 for KNCI.

The fact that the inventory has a measurable and collectible parameter permits the application of parametric tests. The knowledge level parameter was set as the comparison criterion. To test the normality assumption, Kolmogorov‒Smirnov and Shapiro‒Wilk tests, and skewness and kurtosis values were used. The results showed that the total knowledge level in the KNCI did not show a normal distribution. However, apart from these widely used tests, the normality of the data in social sciences is also determined according to the skewness and kurtosis values. The general opinion is that the skewness and kurtosis values between +1.5 and −1.5 in studies with scales such as Likert scale and yes/no scales are sufficient to satisfy the normality assumption. Considering the characteristics of the inventory used in the study and the size of the sample, acceptance limits between +1.5 and −1.5 were preferred for skewness and kurtosis values (33). In further analysis processes, independent samples *t*-tests and analysis of variance (ANOVA) tests were used. Independent Samples *t* Test was used to determine whether the level of knowledge of NC varies significantly with gender, the number of pregnancies, gestational age, the status of having children, mother's work plan, participation in pregnant school, and knowing how to support development. ANOVA test was conducted to check whether the NC knowledge level varies significantly with participants' age, participants' spouses' age, participants’ education level, participants' spouses' education level, and family's income expense status.

## Results

### Descriptive statistics of the sample

The sample of the study, on the other hand, comprised 103 participants, 91 women and 12 men, who were in the 2nd and 3rd trimesters of their pregnancy and volunteered to participate in the study. Descriptive statistics regarding the sociodemographic characteristics of the participants are shown in Table 2.

**Table 2:** Descriptive statistics on the sociodemographic characteristics of the participants.

	*n*	%
**Gender**		
Female	91	88.3
Male	12	11.7
**Participant age**		
18-25 years old	13	12.6
26-30 years old	51	49.5
31-35 years old	29	28.2
36 years old and older	10	9.7
**Participant's spouse age**		
18-25 years old	3	2.9
26-30 years old	47	45.6
31-35 years old	33	32.0
36 years old and older	20	19.4
**Type of place of residence**		
Big city	69	67.7
City	11	10.7
District	18	17.5
Village	3	2.9
Others	2	1.9
**Participant education level**		
Primary-Middle school	3	2.9
High school	4	3.9
University and graduate	96	93.2
**Participant's spouse education level**		
Primary-Middle school	6	5.8
High school	18	17.5
University and graduate	79	76.7
**Year of marriage**		
0-3 years	59	57.2
4 years and more	44	42.8
**Income expense status**		
Equal income and expense	62	60.2
Income higher than expense	25	24.3
Income less than expense	16	15.5
**Total**	**103**	**100**

In terms of gender distribution (Table 2), 88% of the participants were female and 12% were male. In terms of age group, 12.6% of participants were in the 18–25 age group, 49.5% were in the 26–30 age group, 28.2% were in the 31–35 age group, and 9.7% were in the 36 years and over age group. In terms of the age group of participants' spouses, 2.9% of them were in the 18–25 age group, 45.6% were in the 26–30 age group, 32% were in the 31–35 age group, and 19.4% were in the 36 years and over age group. The place of residence had options as large cities, cities, districts, villages, and other settlements. 67.7% of the participants were living in large cities, 10.7% in cities, 17.5% in districts, 2.9% in villages, and 1.9% in other settlements. According to the education level (last grade obtained), 2.9% of the participants were primary-middle school graduates, 3.9% of them were high school graduates and 93.2% of them had university or graduate education. On the other hand, according to the participants' spouses' education level, 5.8% of them were primary school-middle school graduates, 17.5% were high school graduates, and 76.7% had university or graduate education. The duration of their marriage in the time the data was collected was categorized as 57.2% of them have been married for 0–3 years, and 42.8% of them for 4 years or more. In terms of income-expense comparison, 60.2% of them had income equal to their expenses, 24.3% of them had income more than their expenses, and 15.5% of them had income less than their expenses.

In Table 3, the descriptive statistics regarding the pregnancy periods of the people who participated in the study are given. 65% of the participants or their spouses are primigravida and 35% of them are multigravida. When the pregnancy loss status is examined, it is seen that 21.4% of them experienced loss. When the gestational age variable was examined, 46.7% of them were pregnant for 12–24 weeks, and 53.3% of them were pregnant for more than 24 weeks. While the rate of participants stating that their pregnancy was planned was 85.4%, the rate of participants stating that it was not planned was 14.6%. Almost all of the pregnancies (96.1%) were singletons. 35.9% of the babies were girls, while 49.5% of them are boys, and 14.6% of them have not been known yet. 25.2% of the participants have one other child, 1.9% have two other children and 72.8% have no other children. While 26.3% of the participants attended the pregnancy school training, 73.9% of the participants did not. 40.8% of them received child development education, and 59.2% of them did not receive an education.

**Table 3:** Descriptive statistics on the pregnancy characteristics of the participants themselves or their spouses.

	*n*	%
**Number of pregnancies**		
Primigravida	67	65.0
Multigravida	36	35.0
**Pregnancy loss**		
Yes	22	21.4
No	81	78.6
**Gestational age**		
12-24 weeks	47	46.7
25 weeks and more	62	53.3
**Pregnancy plan**		
Planned	88	85.4
Not planned	15	14.6
**State of pregnancy**		
Singleton	99	96.1
Multiple	4	3.9
**Gender of baby**		
Girl	37	35.9
Boy	51	49.5
Not known	15	14.6
**The number of children aside from the current pregnancy**		
One child	26	25.2
Two children	2	1.9
Not any children	75	72.8
**Attending a pregnancy school training**		
Attended	27	26.3
Not attended	76	73.9
**State of receiving education on child development**		
Received	42	40.8
Not received	51	59.2
**Total**	**103**	**100**

### Descriptive statistics related to level of knowledge on NC and its components

Table 4 shows the mean, standard deviation, minimum-maximum, median, mode, skewness, and kurtosis values of the individual knowledge levels of the expectant mothers and fathers on NC and its components. The mean knowledge level of GHC is 9.58 ± 1.33 (out of 13), the mean knowledge level of ANC is 12.55 ± 1.34 (out of 15), the mean knowledge level of RCC is 13.76 ± 1.22 (out of 15), the mean knowledge level of SSC is 10.89 ± 1.14 (out of 12), the mean knowledge level of OELC is 4.34 ± 1.82 (out of 15) and the total knowledge level of NC is 51.14 ± 3.80 (out of 70).

**Table 4:** Descriptive statistics on the level of knowledge on NC and its components.

Component names (Number of questions)	x¯ ± S	Min.	Max.	Median	Mod	Skewness	Kurtosis
Good Health Component (13)	9.58 ± 1.33	6	12	10	10	−0.409	−0,038
Adequate Nutrition Component (15)	12.55 ± 1.34	9	15	13	13	−0.336	−0.181
Responsive Caregiving Component (15)	13.76 ± 1.22	8	15	14	14	−1.577	4.433
Safety and Security Component (12)	10.89 ± 1.14	6	12	11	11	−1.384	2.932
Opportunities for Early Learning Component (15)	4.34 ± 1.82	1	11	4	4	1.123	1.848
Knowledge of Nurturing Care Inventory (70)	51.14 ± 3.80	37	60	52	54	−0.463	0.950

**Table 5:** Results of the independent samples t test and ANOVA conducted to determine whether the participants’ total knowledge levels of NC vary significantly depending on different variables.

	Total Knowledge Level of NC					
	*n*	*x*	*S*	*t*/*F*	Sd Between-groups Within-groups Total sd	*p*
**Gender**						
Female	91	51.57	3.50	3.27	101	0.001*^,#^
Male	12	47.91	4.56			
**Participant age**						
18-25 years old	13	50.69	3.22	0.993	3 99 102	0.399^&^
26-30 years old	51	50.98	4.16			
31-35 years old	29	50.96	3.58			
36 years old and older	10	53.10	3.07			
**Participant's spouse age**						
18-25 years old	3	45.00	7.54	1.531	3 99 102	0.221^&^
26-30 years old	47	50.55	3.50			
31-35 years old	33	51.90	3.76			
36 years old and older	20	52.20	3.03			
**Participant education level**						
Primary-Middle school	3	47.33	2.30	3.481	2 100 102	0.035*^,&^
High school	4	47.75	3.86			
University and graduate	96	51.40	3.73			
**Participant's spouse education level**						
Primary-Middle school	6	48.66	3.26	1.531	2 100 102	0.221^&^
High school	18	50.83	4.14			
University and graduate	79	51.40	3.73			
**Number of pregnancies**						
Primigravida	67	50.86	3.65	-1.018	101	0.311^#^
Multigravida	36	51.66	4.07			
**Gestational age**						
12-24 weeks	41	50.39	3.93	-1.651	101	0.102^#^
25 weeks and more	62	51.64	3.66			
**Having children**						
No	75	50.85	3.62	-1.279	101	0.204^#^
Yes	28	51.92	4.22			
**Mather's work plan**						
Will work	66	50.66	3.90	-1.721	101	0.088^#^
Won’t work	37	52.00	3.52			
**Income expense status**						
Equal income and expense	62	51.53	3.76	0.814	2 100 102	0.446^&^
Income higher than expense	25	50.48	4.12			
Income less than expense	16	50.68	3.49			
**Attending a pregnancy school training**						
Attended	27	52.59	3.34	2.349	101	0.021^*,#^
Not attended	76	50.63	3.84			
**Knowing how to support development**						
Knows	70	51.74	3.66	2.370	101	0.020^*,#^
Doesn’t know	33	49.87	3.84			

^#^ Independent Samples *t* Test.

^&^ ANOVA.

* *p* significance level 0.05.

### Investigation of the level of knowledge of nc in terms of different variables

In Table 5, the level of knowledge of NC was found to vary significantly with gender, participation in pregnant school, and knowing how to support development [*t*(101) = 3.27, *p* < .05; *t*(101) = 2.349, *p* < .05; *t*(101) = 2.370, *p* < .05, respectively]. The mean level of knowledge of the women was found to be 3.65 and higher than that of the men, and this difference is statistically significant. The mean NC knowledge level of those who attended the pregnant school was found to be 1.96 higher than those who did not, and this difference was statistically significant. Similarly, those who stated that they knew how to support development were found to have a 1.87 higher mean NC knowledge level than those who stated that they did not know, and this difference is statistically significant.

The NC knowledge level was found to vary significantly depending on the education level of the participants, *F*(2,100) = 3.481, *p* < .05. This difference was found to be between “those having university and graduate education” and “high school graduates” and “primary-middle school graduates” based on the *post hoc* tests (LSD) conducted. On the other hand, the NC knowledge level was not found to vary significantly depending on participants' age, age, education level and income expense status [*F*(3, 99) = 0.993, *p* > .05; *F*(3, 99) = 1.531, *p* > .05; *F*(3, 99) = 1.531, *p* > .05; *F*(2, 100) = 0.814, *p* > .05, respectively].

## Discussion

The study aimed to determine the knowledge level of expectant mothers and fathers during pregnancy about NC and to examine their level of knowledge in terms of different variables.

### Analysis of the descriptive statistics related to the level of knowledge on NC and its components

The highest mean level of knowledge was found for RCC, with 12.55 ± 1.34 (out of 15), while the lowest level of knowledge was found for OELC, with 4.34 ± 1.82 (out of 15). The mean level of knowledge of the participating expectant mothers and fathers for NC was found to be 51.14 ± 3.80 (out of 70).

In the study, the RCC knowledge level of the expectant mothers and fathers was found to be higher than the knowledge levels found for the other components. When the international literature is reviewed, it is seen that the participants scored higher because RCC is a broad concept, and answers have been sought to the questions asked in the subdimensions such as stress and anxiety and maternal depression related to caregiving skills such as attachment, reciprocity, interaction with the baby, competence in caregiving skills and setting boundaries. For example, in the attachment dimension, it was determined that mothers with a high level of secure attachment, sensitivity, and reciprocity with their babies increased their knowledge to provide better care to their babies (34, 35). It was determined that while the knowledge level of the mothers who received baby massage training during pregnancy increased, their attachment behaviors were also positively affected (36). In a study conducted on mother-infant couples to examine the relationship between the stress and anxiety trajectories of mothers from mid-pregnancy to the postpartum three years and child development at the age of three, it was determined that the mothers who were assigned to the high anxiety symptoms class as a result of multivariate logistic regression analysis had an increased risk of having a child with developmental delay at the age of 3 [adjusted Odds Ratio 2.80, 95% Confidence Interval 2.80 (1.42–5.51), *p* = 0.003] (37). As seen in the research results, the high anxiety and stress of the caregiver primarily result in being sensitive and responsive, which hinders the provision of good health, adequate nutrition, a safe environment, responsive caregiving, and opportunities for early learning, the components of NC, leading to results for children more serious than not reaching developmental potential, such as developmental delays. One of the limited studies examining how different features of maternal depression might be related to the developmental outcomes among low-income children examined two groups of low-income Hispanic immigrant mothers and their children on whether the timing of depression (pregnancy vs. postpartum) and its severity and chronicity are correlated with the cognitive, emotional, and social development of the child (38). While maternal depression was evaluated during pregnancy and at 6 months postpartum, the development of children was followed up to 5 years postpartum. The results showed that maternal depression experienced during pregnancy was correlated with lower cognitive development, especially among girls, while both the timing (pregnancy vs. postpartum) and severity of maternal depression independently were correlated with lower social-emotional development.

These findings highlight the need for early prevention interventions that can be addressed with the NCF to help offset the negative effects of maternal depression on child development in this at-risk population. A qualitative study conducted in low socioeconomic communities of South Africa (19) investigated the facilitators and challengers of effective caregiving in the first 1,000 days through the lens of 30 parents or caregivers. While the facilitators that should be encouraged for effective caregiving were found to range from support systems to professional help for parents, challenging factors were low socioeconomic conditions, and the lack of support from the father to the mother. As effective caregiving is vital in improving developmental outcomes for children in the first 1,000 days of life including pregnancy, there is a need to develop policies and interventions to promote effective caregiving in these first 1,000 days in these communities.

In a community-based comparative study examining the effectiveness of a responsive caregiving-enhancing intervention program (39), the effectiveness of an intervention program educating parents about brain development in the child in the critical first 1,000 days, improving parental self-regulation skills, and teaching the methods of restraint rather than corporal punishment traditionally thought to be an effective practice by parents on the development of children up to the age of 2 years was investigated. It was determined that the children in the intervention group had significantly higher scores in cognitive, fine motor, gross motor, and language development than the children in the control group as a result of the developmental assessments of the children aged two years old. Researchers have found that supporting responsive caregiving, coupled with the knowledge of how development is hierarchically structured, subsequent development depends on early development, how experience in the first year of life alters the plasticity of the brain, and how strong and lasting effects early deprivation has on the brain, can have a significant impact on children's development by complementing the NC approach (39–41).

Although there are no studies in the national literature examining the effect of attachment between mother and baby during pregnancy on the level of knowledge on ECD, many studies have shown that attachment during pregnancy has positive effects in the future (42–46). In this context, it can be said that the high level of knowledge found for RCC in this study is in line with international and national literature.

In this study, it was determined that although the expectant mothers and fathers have a high level of RCC knowledge, their level of OELC knowledge was found to be quite low. A closer examination of the items in both components revealed that the RCC item for which the participants were found to have the highest level of knowledge as all the participants answered it correctly is “*Mother's or father's mental health problems (depression, anxiety disorder, obsessive behaviors) affect the child's development.”* The item for which the participants were found to have the lowest level of knowledge as 25.2% of them gave the wrong answer is “*To communicate with babies, they should not be expected to smile and make eye contact.”* On the other hand, the OELC item for which the participants were found to have the highest level of knowledge with only 31% of them gave the wrong answer is “*At which age do most of the children start walking (taking 5–6 steps unaided) in their lives?*” When the answers of all the participants who gave wrong answers are examined, it is seen that all of them think that the children have gained the ability to walk earlier than the expected period. On the other hand, the item for which the participants were found to have the lowest level of knowledge with %93.2 giving the wrong answer is “*In which month do children smile at the person who is laughing or talking to them for the first time in their lives?”* While this shows that all the parents who gave wrong answers think that this skill can be gained in a time period shorter than the actual time for the mastery of the skill, indicating that the “**opportunity for early learning**” is missed, especially in the field of social-emotional development and that families are in a hurry in the field of movement development expecting their children to master these milestones earlier than their normal time. This false belief of participants overshadows the positive contribution of the abovementioned responsive caregiving to development when supported with early learning and appears to be a factor that will prevent reaching the optimal level in development.

The common finding in international and national studies, the details of which are mentioned below, is the insufficient level of knowledge on early learning and development. Undoubtedly, for OELC, which is a component of the NCF, to be activated, first, the information on development and supporting development must be acquired by parents. From this perspective, while questioning the level of knowledge and research on OELC, the focus is on development and knowing how to support development.

As a result of the studies that questioned the developmental knowledge of mothers in the first three years of life with the same tool in 4 different countries, Turkey, Nepal, Pakistan, and India, it was determined that mothers did not have enough information about when their children achieved developmental milestones and that the limited knowledge they had gained was based on anecdotes. In addition, it was determined that mothers did not have sufficient information about when to start doing activities to support development at the earliest, and therefore, they missed the opportunities for early learning and critical periods for development (12, 14, 17, 47). Mothers who know how to support their children's growth in the earliest period in developmental processes obtain positive results in the long term. While development is supported, impatience, lack of understanding, and seeing the child beyond the expected growth rate lead to negative consequences (48). In a recent qualitative study conducted in Brazil in which the contribution of stimuli presented to children in the first months of life to brain development was investigated, semi-structured questions were asked to mothers. The focus of the mothers was psychomotor development, that they had little knowledge about brain development, and that they did not know how to support brain development. To close this knowledge gap, mothers were given counseling on the issues related to responsive caregiving, such as the importance of senses, social smile, mutual interaction, stimuli, etc., since social development directly affects brain development (49). In another study conducted on 400 Saudi mothers with children up to the age of six, the mothers' general level of knowledge about caregiving and developmental milestones were investigated. It was determined that mothers have limited knowledge of these issues and that the age, education, number of births and pregnancy planning status of the mother affect the mother's knowledge level (16). Another study of Saudi mothers found that while they were knowledgeable enough about certain aspects of child-rearing, especially about physical safety measures, they were not as knowledgeable about the infant's social development (50). Similarly, the results of the study conducted in Qatar to determine the level of child development knowledge of 263 mothers showed that the mothers had poor knowledge of typical child development, developmental norms, and milestones (11).

In a study conducted by Ertem et al. (2007) to determine the knowledge of mothers about child development in Turkey, it was found that most of the mothers believed that developmental skills would be mastered at a later age than they actually are, that their knowledge about motor development was higher and that they did not know the importance of children's social smile and communication (17). The findings of the study conducted by Şahinöz and Bütün Ayhan (2020) in Turkey revealed that 63.7% of mothers do not have enough knowledge about the development of their children (18).

The fact that the question structure of OELC was different from the question structure of the other components may have caused the knowledge level to be lower than expected. However, when compared with the other components examined, the low-level of knowledge on the subjects of development and early learning addressed in OELC is in line with international and national research findings. Thus, in the future, in addition to other components of NC, OELC should be integrated into the programs given to parents and health workers, together with RCC, so that expectant parents or other caregivers can respond sensitively and be aware of their children's development, risks and abilities.

### Analysis of the level of knowledge on NC in terms of different variables

Gender (expectant mother/father), education level, attending a pregnant school, and knowing how to support development were found to be in a statistically significant correlation with the level of NC knowledge. Closer examination revealed that the expectant mothers compared to the expectant fathers, the expectant mothers and fathers who have university and graduate education compared to the expectant parents with lower education, those who attended the pregnant school compared to those who did not attend, and most importantly, those who know how to support development compared to those who do not know have a higher level of knowledge on NC, including all the components.

In relation to the gender variable, in a study conducted on children and their families in 38 LMICs with the purpose of determining the frequency of play and learning activities, it was concluded that 47.8% of the fathers did not engage in any activity with their children, only 6.4% did activities with their children and the ratio of mothers not being engaged in any activity with their children was found to be 21.5%. In the same study, it is also among the results that the activity does not have a positive effect on learning unless it is at a certain level of intensity. These results reveal the necessity for both mothers and fathers to understand, adopt and implement NC (51). Another study examined 181 low-income fathers' knowledge of child development (self-perception and objective) as a predictor of father involvement with infants (verbal stimulation, caregiving, and physical play). The findings suggest that it may be particularly beneficial to enhance fathers' knowledge of child development as well as increase fathers' confidence in their ability to understand and meet their children's needs (52).

A comprehensive study by McCoy et al. (2018) involving children from the low, middle, and high-income countries to investigate the effect of the education level variable reported that the most important factors affecting early learning were the education level of the mother and the stimuli offered to the child in the home environment (53). When two studies conducted in Turkey were examined in terms of the related variables, it was found that mothers with higher education levels and fewer children had higher developmental knowledge because they had more time to spend with their children (17). In another study, it was found that the level of knowledge of the mothers who read using various sources was higher than that of those who did not read, that the educational status of the mother and the number of children they had did not make a difference in terms of developmental knowledge, that low-income status and low education level increased the anxiety level of mothers, and that the level of anxiety decreased as the education level increased (18).

The data of the study aiming to determine the impact of the NCF on the development of more than six thousand children under the age of five living in a poor area of Brazil revealed that a positive socioeconomic status, breastfeeding, lack of strict discipline, presence of caregivers providing responsive caregiving and provision of the opportunities for early learning are the key factors that increase the likelihood of a child reaching his/her developmental potential, even in an upper-middle-income country such as Brazil. These findings provide important insights into which ECD-related policies and programs can be planned, implemented, and disseminated in Brazil and other countries with similar characteristics (54). In a study conducted in Portugal, approximately 500 noncouple parents, in their thirties, of both sexes, with children aged 2–6 years, were examined for the relationship between developmental knowledge and the enjoyment of their caregiving. The results show that parental knowledge of child development predicts caregiving enjoyment (55). These risk factors are also supported by the results of larger international studies. McCoy et al. (2016), in their analysis covering 35 LMICs, examined the developmental data of approximately 99 thousand children aged 3–4 years using the Early Childhood Development Index and determined that 14.6% of the children had low index scores in cognitive, 26.2% in socioemotional domains, and 36.8% in both domains (56). Positive correlations were found between low developmental index scores and body weight z scores below 2 standard deviations compared to height, poverty, male gender, rural residence, and lack of cognitive stimuli. As a result of the evaluations of approximately 12,000 children in Japan when they started the first grade, the effects of the coexistence of multiple dimensions of poverty among children were examined. It was reported that at least one out of every five children experiences financial deprivation, life-related deprivation, or child-related deprivation (lack of stimuli such as children's books, etc.), while 1.4% of children experience these three deprivations together, thus falling behind their developmental potential, and more importantly, this is not known (57).

Data from 135 Population and Health Surveys and Multiple Indicator Cluster Surveys from 94 countries between 2010 and 2018 were used to identify inequalities in early childhood care and development in LMICs. The results of the analysis revealed that 63% of children under the age of 5 did not suffer from “stunting” or “extreme poverty”, 39% of 3- to 4-year-old children participated in “early care and education outside the home”, and 69% of them had “sufficient stimulation in the home environment”. In most countries, children in urban areas or the richest quintiles of household wealth are on average better off than children in rural areas or the lowest wealth quintile in all four indicators. It was also observed that there was no decrease in inequalities detected in any of the indicators over time. As a result, the researchers stated that it is possible to reduce inequalities in these four indicators to improve ECD, especially in LMICs, by providing equal access to high-quality services that adopt the NCF (58).

Although it was done approximately 30 years ago, the results of the study by Dichtelmiller et al. are striking in terms of proving the effect of parents having knowledge about development (59). In the study conducted on babies born under 1,000 g, which is described as extremely low birth weight, and their mothers, the knowledge levels of mothers about infancy, and the cognitive and movement development levels of babies at 8 months of age were compared. The babies of the mothers with above-average knowledge about infant development scored approximately 1 standard deviation (SD) higher in both cognitive and movement development than the babies of the mothers with below-average knowledge, and this difference was found to be statistically significant (59). Parents' developmental knowledge had an impact on developmental outcomes even in high-risk infants, and the importance of supporting caregivers in development was once again demonstrated. In addition to the studies that draw attention to the positive effect of providing appropriate psychosocial stimuli in early childhood on development (60, 61), it is stated that the lack of stimuli and the lack of appropriate psychosocial stimuli to support child development are the most important causes of developmental difficulties (62–64).

In national literature, it is seen that only 74% of children aged 3–4 years (36–59 months) show typical development in at least three of the four developmental areas, in light of the information obtained by asking mothers in the 2018 Turkey Demographic and Health Survey. When each developmental area is examined separately, it is seen that 86% of the children showed as much development as expected from them in the area of literacy-numeracy skills, 27% in the area of social-emotional development, 4% in the area of learning and 2% in the area of physical development (65). The results of a study conducted to determine the effects of biological and psychosocial risks on the development of children in the first months of life in Turkey with the participation of 2,345 children aged 6–42 months revealed that the child's gender being male, body weight z score being below 2 standard deviations compared to height, presence of a chronic disease, mother's low-level education, mother's stating that she does not do anything to support the child's development, and residing in rural areas or slums were independent risk factors for development (66). These two studies, which reflect the data of our country, prove that the frequency of developmental delays in early childhood is too high to be ignored and reveal the necessity of taking action during pregnancy and the first years of life, which are critical for brain development. In the study carried out by Mustafayev (2019), 7% of the mothers did not either answer the question “*What do you do at home to support your child's learning, development and communication*?” or answered “nothing”. As a result, the determination of the mother's statement that she does not do anything to support the development of her child as an independent risk factor for the developmental delay is a finding that needs to be discussed in more detail considering the mothers who stated that they did not know how to support development during pregnancy in the study (66).

As seen, research is based on the fact that parenting begins with the birth of the child. However, at this time, when we are discussing the effects of epigenetics on the development of generations, the pregnancy period is a very valuable opportunity to inform and support expectant parents about the NCF without ignoring OELC since there is a greater knowledge gap in this component. In the international literature, the negative impact of the pandemic mitigation strategies applied during the COVID-19 pandemic, which we have not yet left behind, on the mental health of pregnant women in many parts of the world is mentioned, and it is stated that this is a great concern for pregnant women in caregiving (67). When the national literature is examined, it is seen that among the results of a study by Gürel et al. (2006) in which pregnant women's state of obtaining information in the antenatal period about pregnancy, childbirth, and postpartum periods were examined, women with high education levels were found to have more advanced knowledge than women with low education levels (68). In a study conducted by Kahraman et al. (2016), the knowledge levels of pregnant women about infant care were examined, and it was determined that the level of correctly known knowledge increased as the level of education and age increased (69). Similarly, in the study conducted by Yenal, Okumuş, and Sevil (2010) to define the subjects that pregnant women want to receive counseling about in an interactive environment, it was found that interactive education became widespread due to the improvement in the working rates and living conditions of pregnant women as the education level increased and that the most popular subject was maternal and infant health (70).

When the literature is examined in this context, the results within the scope of the NC overlap with each other, and the literature consensually points out that the concept of NC will contribute to the development and human life starting from the prepregnancy period and in the later years of life and that improvements in economic and health capitals to be invested in disadvantaged groups in early childhood will positively affect these groups in the future (25, 66, 71–74).

All the findings examined throughout the discussion shed light on the fact that it is more likely to meet these needs by implementing the NCF, which conceptually gathers the basic needs of children under a single roof, starting from the pregnancy period.

The main strength of this study is that it addresses the NCF, which is being discussed in the international literature but is a rarely discussed topic in the national literature. While studies on this subject are mostly carried out with parents, working with expectant parents in this study is another important aspect of the study. There are also limitations of this study. The study was limited to expectant mothers and fathers who were into 4–9 months of their pregnancy. The data are limited to the attributes measured by the KNCI and the sociodemographic data form, as NC is such a broad umbrella concept. The expectant mothers and fathers are generally highly educated and have the media literacy to fill out the data form. This may limit the generalizability of our findings to other LMICs with lower education. In line with the measures taken during the pandemic, the inability to conduct face-to-face interviews with the expectant mothers and fathers who are in the high-risk group, and the limited sampling are other important limitations.

## Conclusions

It was determined that the expectant mothers and fathers who participated in the study have the highest level of knowledge on RCC and the lowest level of knowledge on OELC among all the components of NC. In terms of having the highest level of knowledge, RCC is followed by ANC, SSC, and GHC. The general knowledge level of NC of the expectant mothers and fathers participating in the study was found to vary significantly depending on gender in favor of the mothers, education level in favor of those having university or graduate education, attending a pregnant training school in favor of those who attended and knowing how to support the development in favor of those who know how to do it.

In conclusion, it was determined that the expectant parents have information and support needs in all the components of NC and particularly in OELC, which means inquiring about in which period children can achieve basic developmental milestones and what developmental risk indicators are. More research is needed to implement the NCF, especially in LMICs, starting from the pregnancy and prenatal period, so that the opportunities are not missed in early childhood, the period when brain architecture is shaped, and brain development is the fastest. Moreover, the NCF should be given more weight and should be detailed more for supporting children with developmental difficulties, health problems, and/or needing hospitalization, institutional care, and the children rescued from a crisis, conflict, disaster environments, and displaced children as well as supporting their mothers, especially their fathers and other family members, for their participation in supporting the child.

## Data Availability

The original contributions presented in the study are included in the article/Supplementary Material, further inquiries can be directed to tugba.karaaslan@gmail.com.
